# Supplementary material for: The Anti-Leishmania amazonensis and Anti-Leishmania chagasi Action of Copper(II) and Silver(I) 1,10-Phenanthroline-5,6-dione Coordination Compounds
Source: Pathogens. 2023 Jan 1;12(1):70. doi: 10.3390/pathogens12010070 (PMC9865435; doi:10.3390/pathogens12010070)
Supplement: Supplementary file 1 [file pathogens-12-00070-s001.zip › pathogens-2020034-supplementary.pdf]

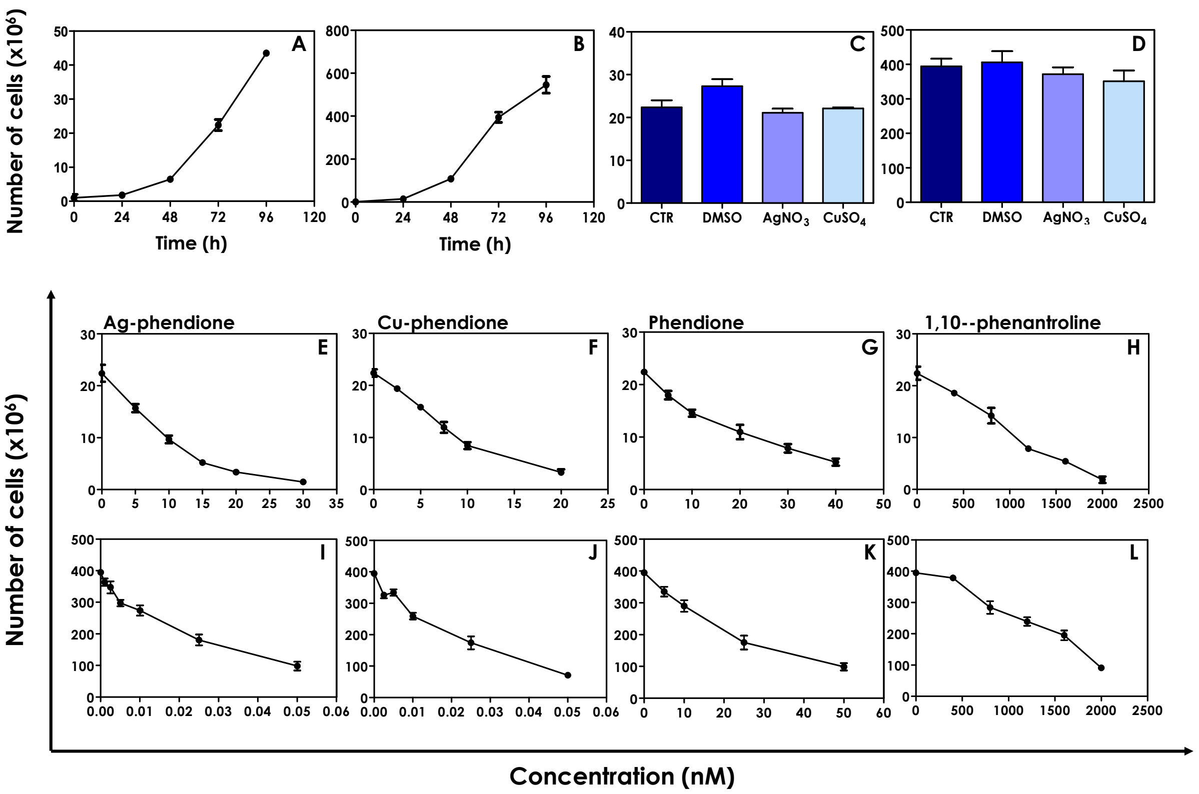

Figure S1: ***Leishmania amazonensis*** **(A) and** ***Leishmania chagasi*** **(B) proliferation profile**. Cells were cultured for 96h in Schneider's medium supplemented with fetal bovine serum and counted daily. **(C-D) Effect of DMSO solvent, and $AgNO_3$ and $CuSO_4$ salts on the proliferation of** ***L. amazonensis*** **(C) and** ***L. chagasi*** **(D).** DMSO was added to the culture in the volume corresponding to the highest concentration of the drug used and the salts in the volume and value corresponding to the highest concentration used in the compounds. **(E-L) Effect of metallocompounds, phendione and 1,10-phenanthroline on the proliferation of** ***L. amazonensis*** **and** ***L. chagasi***. Cells of *L. amazonensis* (E,F,G,H) and *L. chagasi* (I,J,K,L) were cultivated in the absence (CTR) or in the presence of the compounds Cu-phendione, Ag-phendione, phendione and 1,10-phenanthroline, in different concentrations, at 28°C. Each compound was added to the culture on day zero and the cells were counted in a Neubauer chamber after 72 h of incubation.
